# Florid Pseudosarcomatous Polypoid Fibroplasia: An Unusual Cutaneous Manifestation of Chronic Lymphedema

**DOI:** 10.1177/10668969251399912

**Published:** 2025-12-15

**Authors:** Alexander N. Perez, Jonathan L. Curry, John E. Madewell, Jeanne M. Meis

**Affiliations:** 1 Departments of Pathology, Bone and Soft Tissue Pathology Sections, 4002 The University of Texas MD Anderson Cancer Center, Houston, TX, USA; 2 Departments of Pathology, Dermatopathology, 4002 The University of Texas MD Anderson Cancer Center, Houston, TX, USA; 3 Diagnostic Radiology, 4002 The University of Texas MD Anderson Cancer Center, Houston, TX, USA

**Keywords:** pseudosarcoma, lymphedema, cutaneous sarcoma, atypical fibroepithelial polyp, fibroplasia

## Abstract

Bizarre, reactive fibroblastic proliferations associated with localized lymphedema may arise with chronic condom catheter use. We report a unique presentation of innumerable, cutaneous nodules occurring in chronic lymphedema of the right lower extremity due to remote pelvic lymph node dissection and radiation therapy for squamous carcinoma of the uterine cervix. Histologically, the cutaneous nodules consisted of several bizarre, enlarged polygonal to stellate, multinucleated fibroblasts having variably vacuolated cytoplasm in a background of markedly edematous dermis with prominent vascular ectasia. Nuclear hyperchromasia, scattered mitotic figures and pleomorphism in the context of chronic lymphedema led to an original diagnosis of malignancy. However, on histologic re-review, close correlation with imaging studies revealed complete continuity of massive subcutaneous lymphedema with the overlying skin nodules. The presence of diffuse reactive changes of edema and ectasia associated with multinucleated fibroblasts enables recognition of this pseudosarcomatous lesion. To our knowledge, this is the first report of extreme, multiple, cutaneous polypoid lesions comprising bizarre fibroblasts that simulate sarcoma and occur in the setting of chronic lymphedema. Awareness of this entity is critical, as it is easily misdiagnosed as sarcoma complicating lymphedema. We propose the descriptive term florid pseudosarcomatous polypoid fibroplasia for such extreme lesions.

Pseudosarcomatous changes in fibroepithelial polyps of the skin are rarely observed, with only single case reports and small series in the literature.^1,2^ Similar pseudosarcomatous changes are more commonly seen in fibroepithelial stromal polyps of the vulvovaginal region of women and in lymphedematous fibroepithelial polyps occurring in men with use of long-term condom catheters.^3–5^ The occurrence of multiple large pseudosarcomatous nodules in a lymphedematous extremity is extraordinarily rare and poses a diagnostic challenge, with a range of differential diagnoses that includes melanocyte and squamous cell-derived processes, as well as entities such as pleomorphic fibroma, atypical fibrous histiocytoma, and undifferentiated pleomorphic sarcoma (UPS).^6,7^

Herein, we describe multiple nodular skin protuberances occurring in a 59-year-old woman with chronic lymphedema of the right lower extremity. Histologic examination of one of the dermal nodules revealed a cellular proliferation of pleomorphic, bizarre, multinucleated fibroblasts in a background of edematous to hyalinized stroma. The clinical course and radiologic features of these skin nodules support their true reactive nature and do not support a diagnosis of sarcoma or any other malignancy. Morphologically, these lesions share features with other pseudosarcomatous fibroepithelial polyps of the skin or other mucosal body sites, albeit showing florid pleomorphism, which in the setting of extreme lymphedema, led to an initial diagnosis of malignancy.

## Case Report

**Figure 1.**
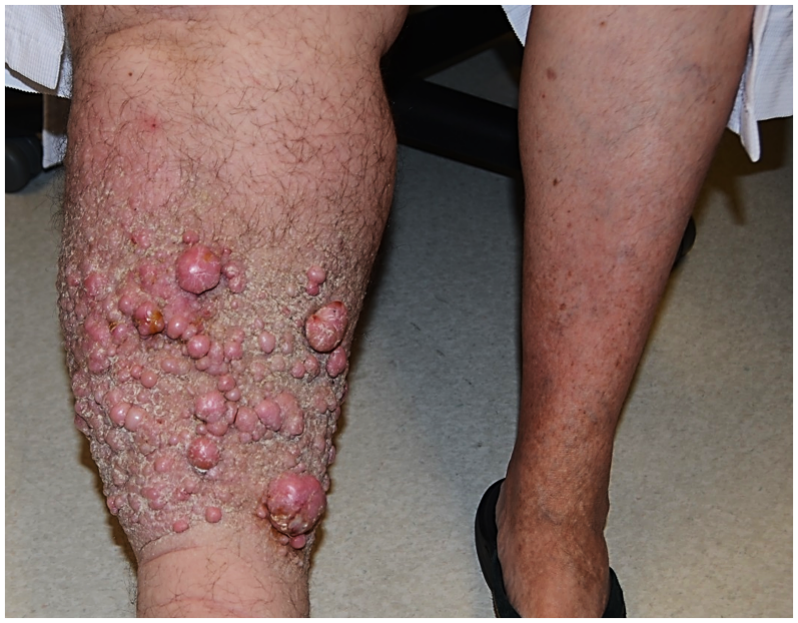
Clinical photograph of the patient's affected (right) lower extremity. There is marked lymphedema with innumerable polypoid, fleshy protuberances in the mid-shin.

**Figure 2.**
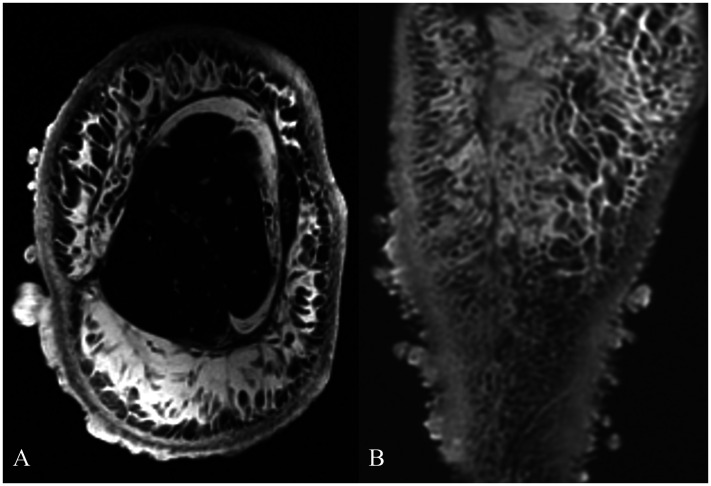
Radiologic imaging studies. A) Axial (T1-weighted image) revealing edema infiltrating subcutaneous fat and skin, extending down to the level of the deep fascia. the underlying bone and skeletal muscle show no significant alterations. B, Coronal (T1-weighted image, after injection of gadolinium contrast) revealing extensive edema with thickened skin and prominent subcutaneous vasculature. Note prominent dermal nodules.

**Figure 3.**
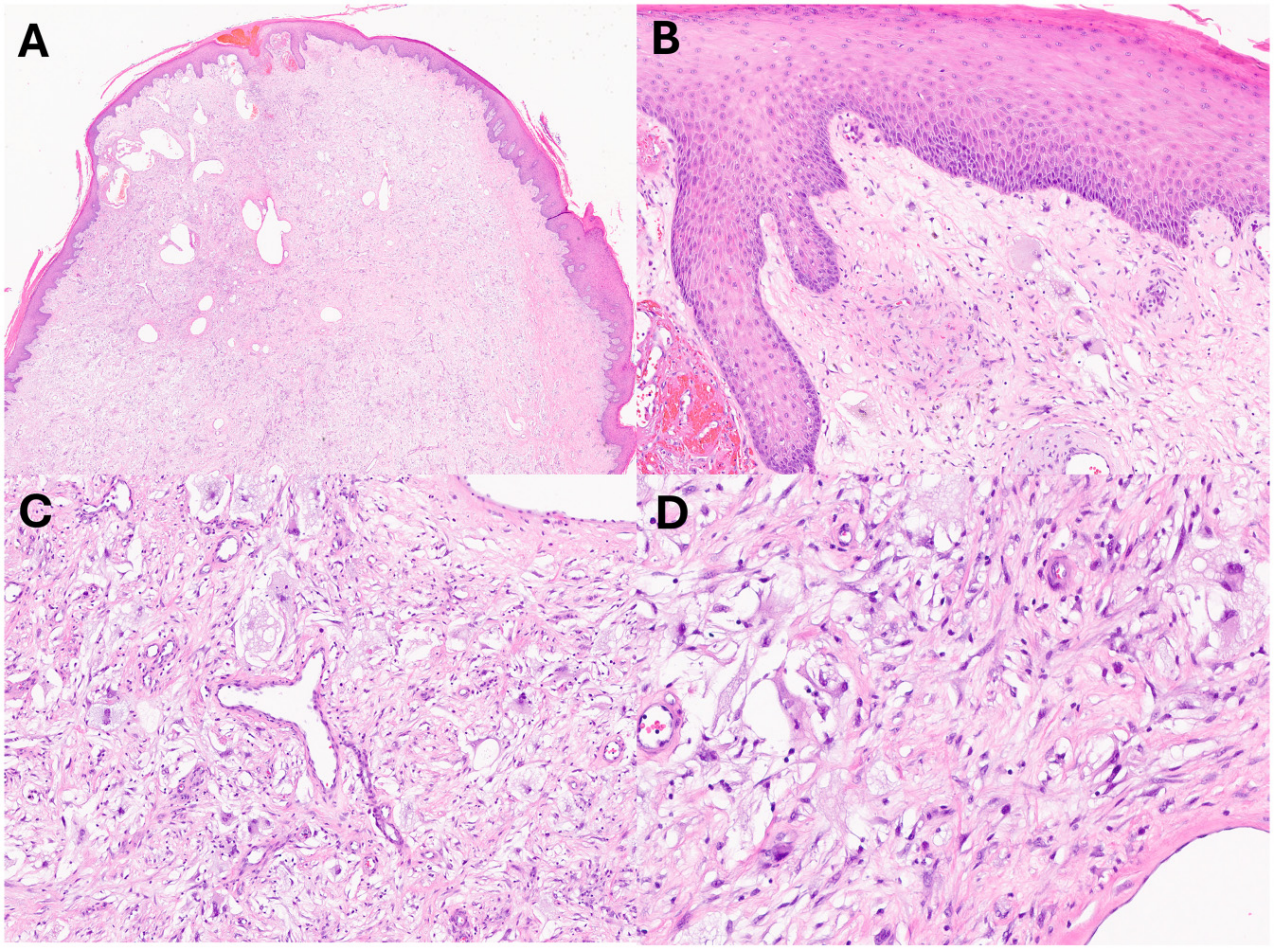
Histologic images of cytomorphology. The lesion is polypoid as seen on lower magnifications (Hematoxylin and Eosin (H&E), A, 20x and B, 100x). The lesion is composed of spindled and giant cells (H&E, C, 100x). Focal areas showed predominance of myofibroblast-type spindle cells (H&E, D, 200x).

**Figure 4.**
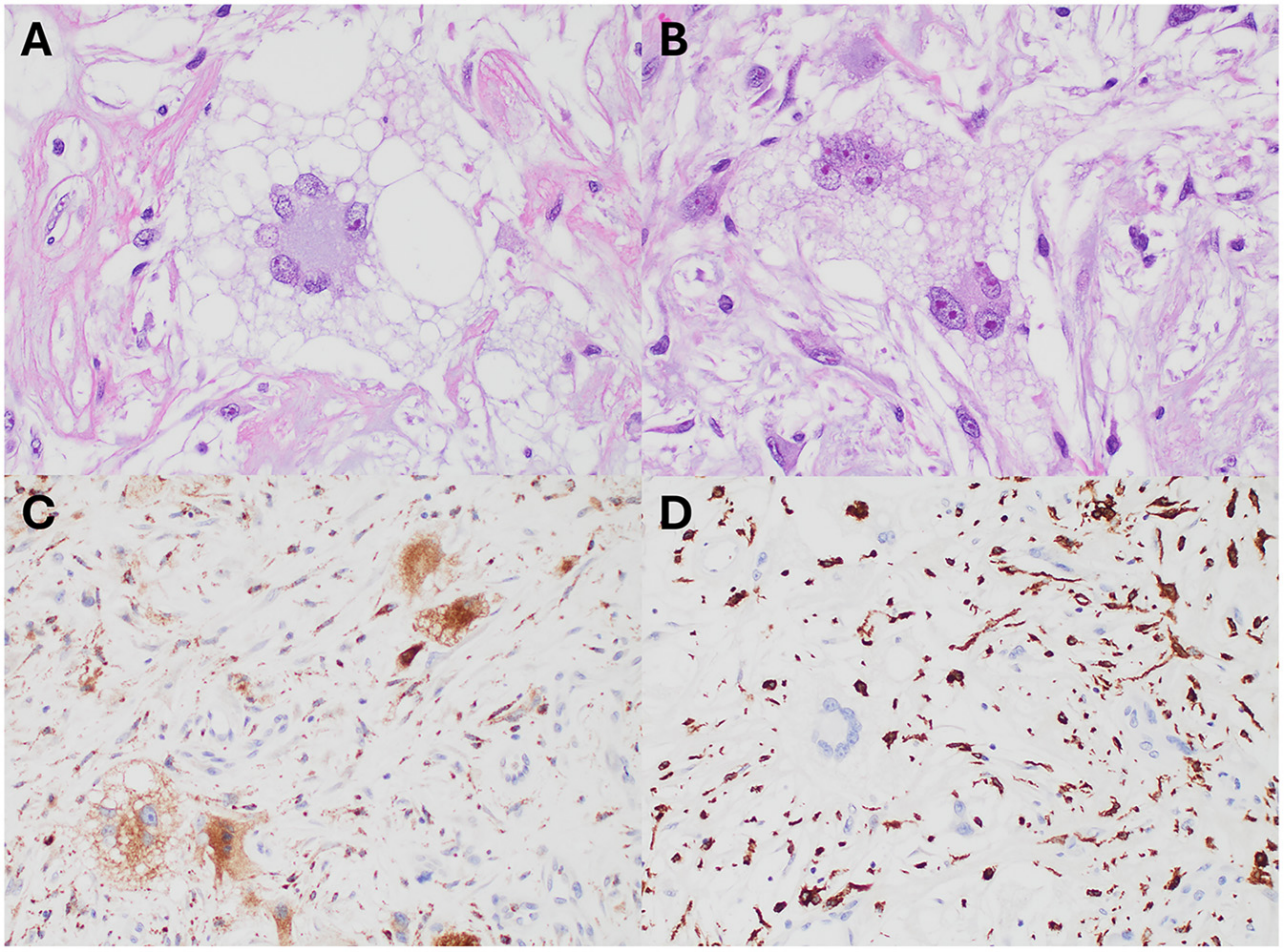
Histologic and immunohistochemical images. the multinucleated giant cells showed variable morphologies, with touton-type forms (Hematoxylin and Eosin (H&E), A, 400x) and more conventional-type giant cells (H&E, B, 400x). the nuclear features were concerning due to hyperchromatic, vesicular chromatin and macronucleoli. The spindle cells also show similar nuclear details. Immunohistochemical (IHC) stains show the multinucleated giant cells express CD68 (IHC, C, 400x) and the spindle cells express CD163 (IHC, D, 400x).

A 59-year-old woman with a history of stage IB squamous carcinoma of the uterine cervix 20 years prior, status post radical hysterectomy with bilateral salpingo-oophorectomy, bilateral pelvic, lymph node dissection and adjuvant seed radiotherapy, subsequently developed advanced lymphedema of the right lower extremity. She noticed the development of skin nodules 18 months after her surgery, and presented to a plastic surgeon with multiple protuberant, pedunculated dermal nodules 2 years postoperatively (Figure 1A). Magnetic resonance images of the lower extremity revealed massive lymphedema of the subcutaneous adipose tissue in direct continuity with innumerable protruding skin nodules (Figure 2A-B). Biopsy examination of one of the skin nodules demonstrated a cellular dermal infiltrate of atypical, pleomorphic cells in an edematous to mildly fibrotic stroma, associated with vascular ectasia (Figure 3A-D). Scattered cells were multinucleated with hyperchromatic nuclei and abundant, foamy cytoplasm (including occasional Touton-like forms) (Figure 4A-B). Mitotic figures, including rare, atypical forms, were identified.

Immunohistochemical studies revealed that the atypical giant cells were positive for CD68 and negative for CD163. Adjacent smaller dermal infiltrates of histiocytes were reactive for CD163 (Figure 4C-D). Ki-67 and PHH3 highlighted areas of proliferation, less than 5% and scattered mitotic figures, respectively. The atypical cells were negative for a keratin cocktail (keratins AE1/AE3, 8/18, CAM 5.2 and MNF 116), CD31, CD34, and SOX10, thereby excluding epithelial, vascular, and neural crest origin. C-MYC was largely negative.

The initial diagnosis was undifferentiated pleomorphic sarcoma (UPS), but on histologic review for a multidisciplinary tumor board, the diagnosis was modified to a pseudosarcomatous proliferative fibroplasia secondary to lymphedema. This modified diagnosis was further supported by the MRI findings, which showed massive lymphedema of the subcutaneous adipose tissue of the lower extremity in direct continuity with the skin nodules (Figure 2).

The patient underwent surgical management 9 months following the initial sampling, which included excision of multiple nodules (∼50, size range 1 to 10 cm) from the lower extremity. Additionally, omental vascularized lymph node transfer and lympho-venous anastomosis were performed, necessitating a split thickness skin graft. Histological sampling of post-treatment dermal nodules revealed involution of the atypical fibroplasia and generalized dermal sclerosis with nearly complete resolution of the vascular ectasia. At 3 months follow-up, there was marked improvement of the patient's lymphedema. However, the patient gradually re-developed lymphedema without formation of polypoid skin nodules over the next 4 years, complicated by skin infections requiring long term antibiotic treatment. During surveillance, an incidental retroperitoneal schwannoma was identified and biopsied.

## Discussion

Although infection is a major cause of lymphedema worldwide, in developed countries, it is more commonly a therapeutic sequela. Less common causes include congenital malformations and obesity.^8,9^ Chronic lymphedema may be further complicated by the development of nodules or masses, which can in turn arouse suspicion of malignancy.

By light microscopy, the histologic features of chronic lymphedema reflect the underlying pathogenesis, with dilated, thin-walled dermal vascular spaces and edema in the surrounding dermis resulting from lymph extravasation. Variable inflammatory infiltrates may be seen. Chronic lymphedema also produces accentuation of subcutaneous fibrous septa.^9^

Malignancy arising within lymphedema is well documented. Angiosarcoma often arises in this setting, having been first described by Drs. Stewart and Treves in 1948 in their seminal series of patients with angiosarcoma several years after treatment for breast carcinoma.^10^ As lymph node dissection and radiation therapy have increased in the treatment of various malignancies, chronic lymphedema and a secondary malignancy have become potential long-term complications. Angiosarcomas have been described in the lower extremities of patients with gynecologic malignancies who had pelvic lymph node dissection and/or radiation therapy.^11,12^ Additionally, patients with severe obesity have been documented to develop massive lymphedema, and angiosarcoma has been identified in this population in the absence of surgical instrumentation or radiation.^13^

In contrast to the discolored and plaque-like lesions of angiosarcoma, polypoid/protuberant lesions within the background of chronic lymphedema are uncommon. The latter more typically occur as fibroepithelial polyps, most frequently in the lower genitourinary tract (of both women and men), and have been reported in the setting of acquired and congenital lymphedema.^4,12^ In a case report of a vulvar lesion by Orosz et al,^14^ the histologic findings were of a markedly edematous lesion, with many dilated vessels and intervening hypocellular bland, fibroblastic-appearing cells (supported by the immunohistochemical finding of smooth muscle actin reactivity). Only rare, atypical multinucleated cells were present. There was concern for sarcoma after development of two recurrences (1 and 6 years). Our specimen had similar histologic features and extensive sampling of the reconstructive procedure specimens had no evidence of malignancy.

Pseudosarcomatous fibroepithelial polyps are rare lesions. Review of the medical literature yielded few examples outside of the genital region.^15–17^ The first report of such, by Williams et al (1996), described two patients with similar polypoid lesions of long (∼20 year) duration.^1^ These lesions arose on the leg and the back, and were both skin-colored, fleshy protuberances. No mention was made of clinical lymphedema. Histologic examination showed acanthotic epidermis, with dilated dermal vasculature (including some with hyalinization) and similar large, hyperchromatic, stellate cells as seen in our reported lesions. More recent series, by Cathro et al^15^ and Potts et al^16^ have reported similar lesions under varying nomenclatures, including cutaneous pseudosarcomatous polyp and pleomorphic fibroma. None of these have known local recurrence or metastasis, and all lesions were solitary.

The histologic differential diagnosis for these lesions is broad, including entities of epithelial (such as spindle cell/sarcomatoid squamous cell carcinoma) and melanocytic (including intradermal nevus and/or nevoid melanoma) origin, which are readily excluded with appropriate immunohistochemical stains. The lesions we describe show features of modified fibroblasts, albeit bizarre features, raising suspicion of sarcoma. Depending on the clinical setting, diagnostic considerations include undifferentiated pleomorphic sarcoma, giant cell fibroblastoma, aggressive angiomyxoma, superficial CD34 + fibroblastic tumor, spindle cell/pleomorphic lipoma, and others.

**Figure 5.**
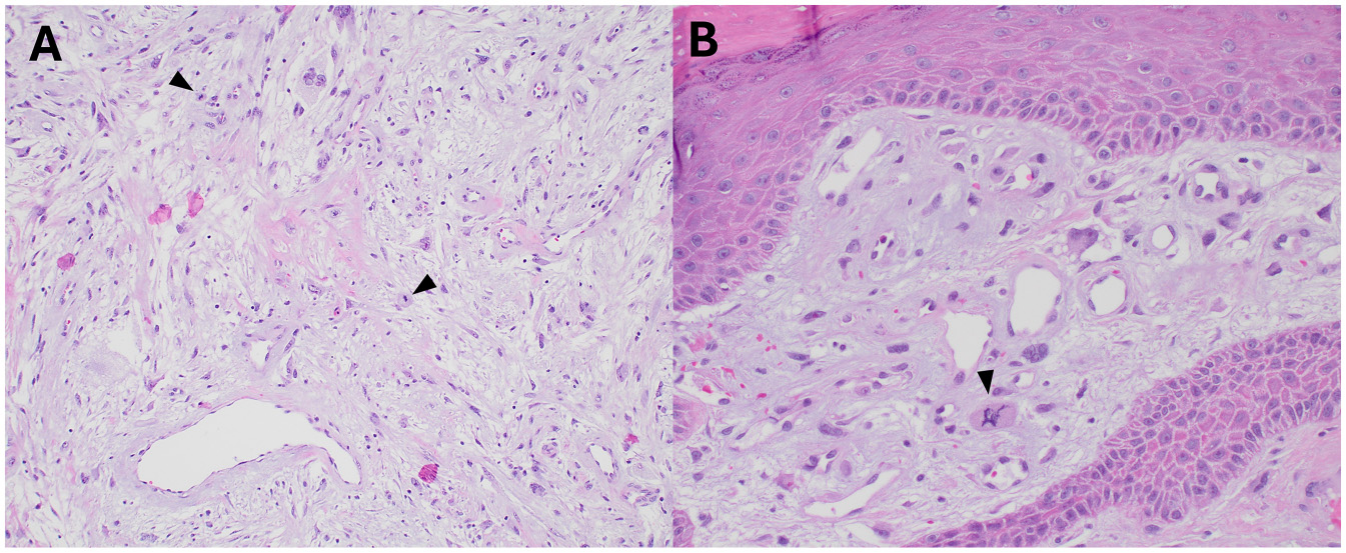
Histologic images showing proliferative activity (including atypical forms). Mitotic figures were scattered within the lesion (H&E, A, 100x, arrowheads). Rare, apparent atypical forms were also identified (H&E, B, 200x).

The morphologic features of the lesion we describe are of a mild-moderately cellular proliferation composed of spindled cells with vague fascicular and storiform arrangements. The tumor nuclei were hyperchromatic and vesicular, with variably dispersed chromatin. Scattered multinucleated stromal cells were noted, some with Touton-like features. The stroma was eosinophilic, fibrillary, and loose, with fibrillary collagen. The superficial aspect of the dermis showed markedly dilated and thin-walled vessels, reflecting lymphangiectasia and somewhat reminiscent of angiokeratoma. As described earlier, the immunophenotype was not specific, and excluded several lines of differentiation. Significant mitotic activity was not seen, which was corroborated by minimal expression of proliferation markers (Ki-67 and PHH3). However, rare, atypical (multipolar) mitotic figures were identified Figure 5). While typically associated with malignant neoplasms, this finding has been described by others in atypical decubital fibroplasia / ischemic fasciitis.^17^

To the best of our knowledge, this is the first case report of florid pseudosarcomatous nodular proliferations of the leg in the setting of massive chronic lymphedema. The diagnosis of a reactive process in this instance was confirmed by correlation with radiologic images. The striking appearance of innumerable nodules in a patient with marked changes of lymphedema by MRI would be unusual for any of the previously mentioned sarcomas or benign mesenchymal tumors mentioned in the differential diagnosis.

In our view, this is a distinctive lesion having unique clinical and pathologic features which is best described by the term “florid pseudosarcomatous polypoid fibroplasia” (FPPF). This terminology encapsulates the notion of fibroblastic hyperplasia with bizarre forms that may cause confusion with malignancy, particularly when the radiologic or imaging findings are not known. Of note, the lesions did not recur over a period of four years after a surgical lymphatic flap procedure to correct the lymphedema. However, as the underlying cause for lymphedema cannot be remedied entirely by surgery, complications (such as multiple episodes of infection as seen in this patient) or the development of subsequent similar skin nodules remain potential complications, as does the development of a sarcoma. This particular presentation illustrates the need for long-term follow-up in patients with chronic lymphedema, re-evaluation of the extent of radical lymph node dissections, and the pro-active development of earlier interventions to prevent the development of secondary complications of lymphedema.
